# Public views on religious and financial care restrictions in hospitals

**DOI:** 10.1093/haschl/qxag015

**Published:** 2026-01-25

**Authors:** Cooper Urban, Cory Cronin, Samuel Doernberg, Ria Dharnidharka, Lauren Taylor

**Affiliations:** Department of Population Health, NYU Grossman School of Medicine, 180 Madison Avenue, New York, NY 10016, United States; Department of Social and Public Health, Ohio University's, Grover Center, W324, Athens, OH 45701, United States; Ohio University Appalachian Institute to Advance Health Equity Science (ADVANCE), Athens, OH 45701, United States; Department of Medicine at NYU Grossman School of Medicine, 550 1st Avenue, New York, NY 10016, United States; Department of Population Health, NYU Grossman School of Medicine, 180 Madison Avenue, New York, NY 10016, United States; Department of Population Health, NYU Grossman School of Medicine, 180 Madison Avenue, New York, NY 10016, United States

**Keywords:** hospital ethics, public opinion, health care access, health literacy, care restrictions, religious affiliations, hospital finances

## Abstract

**Background:**

Hospitals make decisions about which services to provide based on a variety of factors. However, decisions to provide services based on financial or religious considerations have increasingly drawn public scrutiny. We conducted a national survey to assess public attitudes toward hospitals’ financial or religious motivations for offering certain types of care.

**Methods:**

We conducted a national, cross-sectional online survey of 1577 US adults. Respondents indicated on a 3-point, frequency-based Likert scale whether hospitals should “Never,” “Sometimes,” or “Always” be allowed to limit services for these reasons. Descriptive statistics and multivariable logistic regression analyses examined the demographic and experiential correlates of these attitudes.

**Results:**

Most respondents opposed financially motivated restrictions (62%), while a plurality opposed religiously motivated restrictions (48%). Opposition differed across subgroups, with Republicans, individuals with public insurance, and health care employees more accepting of both types of restrictions, while older respondents and those with higher health literacy were more likely to oppose them.

**Conclusion:**

Our findings reveal a notable divergence between how hospitals often operate and what the public believes hospitals should be permitted to do. Efforts to improve transparency around service limitations and ensure continuity of care may help maintain public trust when hospitals decline to provide certain services.

Key Takeaways

- Public opposition to limiting services for financial and faith-based reasons is widespread.
- Financially motivated limitations on hospital care face stronger opposition than religious ones.
- Republicans, individuals with public insurance, and health care workers were more accepting of such limitations, whereas those with higher health literacy and older adults were more opposed.

## Introduction

Hospitals occupy an unusual space in the public realm, serving as essential public touchpoints for vital medical care while largely remaining privately governed institutions. This structure affords hospitals considerable discretion over many aspects of their operations, including routine choices about how to allocate space, staff, and resources. Some of these choices may spark more controversy, including decisions about which services to maintain and which to discontinue, independent of public need or demand. These decisions can alter the availability of clinical services and become highly visible and contested, particularly when they shape access to care in ways that the public perceives as ethically or socially significant.

Some hospitals use available discretion to provide services that extend beyond conventional medical care, such as clinical trials, high-touch care coordination, food pantries, acupuncture, or valet parking. That same discretion, however, can also lead hospitals to restrict other services. In some cases, hospitals limit services that many consider to be within the standard scope of health care. Financial strategy or the moral and religious commitments of the institutions themselves are primary drivers of these limitations. Much of hospital decision-making around service offerings occurs with little public visibility. Nevertheless, individuals may hold normative expectations about what hospitals should be permitted to do, which can be challenged when service restrictions become visible through emergency care encounters, mergers, media coverage, or referral failures.

A substantial minority of US hospitals are religiously affiliated, and their institutional identity shapes the scope of care they provide. Religious health care institutions comprise 16% of all US hospitals and 4 of the 10 largest health systems in the country.^1^ These institutions operate under the Ethical and Religious Directives for Catholic Health Care Services^2^ (ERDs), which guide organizational decision-making on the scope of care they provide. As a result, certain types of care, such as reproductive and end-of-life services, may be limited or only offered under specific conditions. Such policies can influence the availability and timeliness of care in clinically significant situations, including miscarriage management,^3^ treatment of ectopic pregnancies,^4^ and postpartum sterilization.^5,6^ Similar service decisions can arise in other faith traditions as well. For example, some Protestant institutions may offer abortion services only in select situations where the patient's health or fetal viability is in jeopardy, or in cases of rape or incest.^7^ Prior work suggests that these institutional differences may extend beyond service availability to differences in patient outcomes, including neonatal outcomes,^8^ hospital performance metrics,^9^ and reproductive care experiences in religiously affiliated vs secular hospitals.^10^ However, far less is known about how the public evaluates hospitals’ use of discretion when such restrictions are motivated by religious or financial considerations.

Other service decisions are made for financial reasons. While some hospitals may have never offered certain services because they are not financially viable, others choose to discontinue services they previously provided. Some discontinuations reflect strategic efforts to maximize margins, such as when low-revenue services are cut or higher-revenue services are expanded following acquisition by private equity firms. For example, hospitals acquired by private equity firms may discontinue operations that primarily serve uninsured or Medicaid patients in favor of higher-revenue lines of care.^11^ Other hospitals may be forced to discontinue services out of financial distress rather than margin-maximization.^12-14^ For instance, insufficient reimbursements from public health insurance programs such as Medicaid have contributed to closures of rural obstetric units,^12^ and emergency departments (often low-margin for hospitals^15,16^) also face financially motivated closures nationwide.^14^ In general, hospitals that rely heavily on public insurance are particularly vulnerable and therefore more likely to face such decisions.^17^

Media coverage and public discourse may suggest that some people find such non-provision of care troubling, framing it as a violation of a perceived right to care. Yet hospitals operate within an American political tradition that places strong normative weight on both market freedom and religious liberty. These 2 traditions, rooted in longstanding legal protections, provide powerful motivations for hospital discretion over service offerings. We sought to understand how the public negotiates the tension between expectations of access to health care and these deeply held traditional commitments to economic and religious freedom. Through a national survey, we examined respondents’ attitudes about how often hospitals should be permitted to not provide services for financial or religious reasons, thereby highlighting the values that shape public expectations of hospitals as both moral and economic actors.

## Methods

We designed a national, cross-sectional online survey of US adults to assess public views on whether hospitals should be permitted to restrict care for financial or religious reasons. The survey was fielded through NYU Grossman School of Medicine's Qualtrics platform and distributed by Lucid Theorem (now Cint) between January 22 and 28, 2025. Participants were recruited from Cint's managed online research panel. Quotas were applied to approximate the demographic composition of the US adult population with respect to age, gender, race and ethnicity, education, region, and political affiliation, based on US Census data.

To explore whether framing hospitals as businesses influenced responses, a framing experiment was embedded in the survey. Half of participants viewed a brief preamble (Appendix—Figure S1) comparing hospitals to other businesses before answering questions about the permissibility of service non-provision. There were no significant differences between the responses of those who viewed the preamble and those who did not, so responses were combined for this analysis.

Respondents were asked 2 questions about whether hospitals should be permitted to (1) “not offer certain types of care (eg, abortion or gender-affirming care) based on their religious commitments” and (2) “not offer certain types of care (eg, child psychiatry) because the services are not financially profitable”. Response options were “Never,” “Sometimes,” and “Always.” Additional data on respondents’ demographics and prior health care experiences were collected, including gender, age, race and ethnicity, education, political affiliation, insurance type, rural residence, self-rated health, financial strain, prior mistreatment in health care, past-year hospitalizations, and health literacy.

We report descriptive statistics and multivariable logistic regression models examining associations between respondent characteristics and attitudes toward hospital care restrictions.

## Results

The final sample included 1577 respondents whose demographic characteristics are summarized in Table 1. Overall, respondents were strongly opposed to both religiously and financially motivated care restrictions (Table 2). A plurality indicated that hospitals should never be permitted to restrict care for religious reasons (48%), and a majority expressed this view for financially motivated restrictions (62%). Participants selected “Never” two and a half times as often as “Always” for religiously motivated care restrictions, and more than 6 times as often for financially motivated restrictions (Table 2).

**Table 1.** Sample demographics.

Demographics	Frequency (%) or mean (SD)
**Gender**	
Woman	847 (53.7%)
Man	692 (43.9%)
Transgender woman	7 (0.44%)
Transgender man	7 (0.44%)
Nonbinary/gender fluid/gender expansive	12 (0.76%)
**Ethnicity**	
Are you of Hispanic, Latino, or Spanish origin?—Yes	241 (15.3%)
Are you of Hispanic, Latino, or Spanish origin?—No	1338 (84.7%)
**Age, y**	
18-39 y	576 (36.4%)
40-59 y	514 (32.5%)
60+	493 (31.1%)
**Educational level**	
Some high school	67 (4.2%)
High school diploma or equivalent	735 (46.3%)
Associate's degree	318 (20.1%)
Bachelor's degree	326 (20.6%)
Graduate degree	137 (8.7%)
**Race**	
American Indian or Alaska Native	42 (2.7%)
Asian	74 (4.7%)
Black or African American	248 (15.7%)
Native Hawaiian or Other Pacific Islander	9 (0.57%)
White	1075 (68.0%)
Others (please give details)	68 (4.3%)
Selected >1	64 (4.0%)
**Rural vs non-rural**	
Rural	476 (30.1%)
Non-rural	1107 (69.9%)
**Region**	
Northeast	276 (18.2%)
Midwest	319 (21.0%)
South	616 (40.6%)
West	308 (20.3%)
**Health insurance**	
I have public health insurance (eg, Medicaid/Medicare/TRICARE)	921 (58.3%)
I have health insurance through my or a family member's employer	339 (21.4%)
I purchase health insurance independently	144 (9.1%)
Other (please give details)	47 (3.0%)
**Political affiliation**	
Republican	526 (33.3%)
Democrat	555 (35.1%)
Independent	427 (27.0%)
Something else	74 (4.7%)
**Health literacy** ^a^	
Always need help understanding	32 (2.1%)
Often need help understanding	61 (3.9%)
Sometimes need help understanding	377 (24.1%)
Rarely need help understanding	455 (29.1%)
Never need help understanding	637 (40.8%)
**Health status**	
Poor	72 (4.6%)
Fair	378 (23.9%)
Good	693 (43.9%)
Very good	321 (20.3%)
Excellent	116 (7.3%)
**Financial stress**	
Yes	520 (33.0%)
No	1055 (67.0%)
**Work in health care**	
Yes—clinical	58 (3.7%)
Yes—operational	35 (2.3%)
Yes—research	22 (1.4%)
Yes—administrative	71 (4.5%)
No	1391 (88.1%)
**Past-year hospital admission (18+)**	
Yes	540 (34.1%)
No	1043 (65.9%)

^a^Health literacy was measured using a single-item screening question asking how often respondents have difficulty understanding written medical information.

**Table 2.** Participant responses to care restriction permissibility questions.

How often should a hospital be able to:	Never	Sometimes	Always
*not offer certain types of care (eg, abortion or gender affirming care) based on its religious commitments?*	48% (n = 769)	33% (n = 521)	19% (n = 309)
*not offer certain types of care (eg, child psychiatry) because they won't make the hospital enough money?*	62% (n = 985)	28% (n = 446)	10% (n = 162)

Multivariable logistic regression models (Appendix Table S1) indicated that attitudes toward care non-provision differed across demographic and experiential groups. Older adults and respondents with higher health literacy were more likely to indicate that religiously motivated restrictions should never be permitted than reference groups. Republican respondents and those employed in health care were less likely to oppose religious restrictions.

Opposition to financially motivated restrictions was stronger among women, older adults, and respondents with higher health literacy than their respective reference groups. By contrast, Republican respondents, Black respondents, those with a past-year hospitalization, individuals with public insurance, and health care employees were less likely to oppose financially motivated restrictions.

## Discussion

In this national survey, respondents generally opposed hospitals restricting care for either financial or religious reasons, with stronger opposition toward financially motivated limits on service provision. Although financial and religious restrictions on care are legally permissible and occur frequently in US hospitals, the view that such practices should always be permissible represents a minority perspective among respondents. This contrast points to a fundamental misalignment between common organizational behaviors and public expectations of what hospitals should be allowed to do. When service limitations depart from these expectations, even legally permissible decisions may be seen as violating hospitals’ perceived obligations to the communities they serve. Furthermore, because hospital service decisions are often made with limited transparency, public expectations may be most consequential when restrictions are revealed in moments of clinical urgency or institutional change. In such contexts, understanding public expectations may help anticipate community responses to service reductions and inform how hospitals communicate and justify decisions that limit access to care. This misalignment carries risks for public trust in hospitals, given that transparency, a clear prioritization of patient care over profit, and the consistent delivery of high-quality care are key drivers of patient trust in health care institutions.^18,19^

Although opposition was strong for both financially and religiously motivated restrictions, response patterns differed between them. A plurality of respondents indicated that hospitals should never be allowed to restrict care for religious reasons, while a majority rejected financially motivated restrictions. This distinction is increasingly relevant given that Catholic health systems represent a growing proportion of US hospitals—between 2001 and 2020, the number of Catholic hospitals increased by 28.5% (to 577 in 2020), while non-Catholic hospitals declined by 13.6%^1^ (to 3083 in 2020). The asymmetric reaction suggests that respondents may view faith-based restrictions as different in kind, rooted in organizational identity and moral conviction rather than cold financial calculation. This is consistent with American legal frameworks that afford special protection to religious conscientious objection, while purely financial decisions receive no comparable deference. Our findings echo these distinctions, indicating that the public evaluates the legitimacy of service limitations through the lens of the underlying motivation.

Among the subgroups examined, Republicans stood out as significantly less likely to respond that either motivation for non-provision should never be allowed. This pattern may reflect strong ideological endorsement of market freedom^20^ and greater religiosity,^21^ which in turn could extend to support for hospitals having greater latitude in making religiously based service decisions. Healthcare workers were also less likely to oppose religiously motivated restrictions, which may reflect greater familiarity with institutional policies governing conscientious objection and service provision. Together, these findings build on prior work showing partisan differences in how strongly access to care is viewed as an essential part of health care quality^22^ and suggest that both political orientation and professional experience shape how individuals evaluate hospitals’ discretion to limit services.

The substantial misalignment between common hospital behaviors and public expectations carries practical implications for how hospitals communicate and justify service limitations. When financially motivated service reductions occur, they may be perceived as inconsistent with hospitals’ social obligations, particularly in communities already facing limited access. In contrast, restrictions grounded in religious doctrine may be interpreted through a more familiar framework of conscientious objection, especially when hospitals clearly articulate the commitments informing their policies and ensure continuity of care through referral or transfer.^23^ Across both contexts, transparent communication may be central to maintaining public confidence and legitimacy.

Overall, public attitudes toward hospital care restrictions demonstrate strong expectations that hospitals uphold access to care, with different reactions depending on whether limits stem from financial or religious motivations. These findings highlight the importance of understanding how hospitals’ decisions surrounding service provision are interpreted by the communities they serve. Future research should examine how the public forms expectations about hospital obligations, how expectations may change depending on which clinical services are affected, and how hospitals communicate decisions regarding service availability. Additional work should also assess how such decisions affect patients’ experiences of accessibility and fairness. In an era of growing financial and moral pressures in US health care, strengthening transparency and fostering accountability to community expectations may be central to sustaining hospitals’ legitimacy and ethical credibility.

### Limitations

This study has several limitations. Data collection occurred shortly after a highly publicized, violent, healthcare-related event, which may have influenced respondents’ opinions of healthcare institutions. Additionally, although sampling quotas improved demographic balance, some groups may remain over- or underrepresented. Finally, the survey did not specify a broad range of clinical services or contexts, limiting the ability to further assess whether attitudes vary across types or urgency of care.

## Conclusion

In this national survey, public opinion largely rejected hospitals restricting care based on either financial or religious motivations, with opposition strongest toward financially motivated limits. These views underscore a notable divergence between public expectations and the service decisions hospitals routinely make. Such divergence may weaken public confidence in hospitals’ decision-making, underscoring the importance of transparency and continuity of care when restrictions occur.

## Supplementary Material

qxag015_Supplementary_Data
