# Supplementary material for: Public views on religious and financial care restrictions in hospitals
Source: Health Aff Sch. 2026 Jan 25;4(2):qxag015. doi: 10.1093/haschl/qxag015 (PMC12869792; doi:10.1093/haschl/qxag015)
Supplement: qxag015_Supplementary_Data [file qxag015_supplementary_data.zip › Revised Appendix Materials_HAS.docx]

*Supplemental Material for* **Public Views on Religious and Financial Care Restrictions in Hospitals**

*Table 3 (****Appendix****) – Multivariable logistic regression model of opposition to hospital care restrictions*

| *Table 3. Multivariable logistic regression model of opposition to hospital care restrictions* | | | | |
| --- | --- | --- | --- | --- |
|  | *Not offering certain care based on religious commitments*  *(n=1496)* | | *Not offering certain care based on financial commitments*  *(n=1489)* | |
| **Outcome** | Never count | | Never count | |
| **Model** | Logit | | Logit | |
| **Predictors** | OR | p-value | OR | p-value |
| Female | 1.225 | .071 | 1.378** | .008 |
| Age 18 - 39 | 1.000 |  | 1.000 |  |
| Age 40 – 59 | 1.456** | .007 | 1.425* | .013 |
| Age 60 + | 1.462* | .014 | 2.922*** | .000 |
| White | 1.000 |  | 1.000 |  |
| Black | .877 | .431 | .675* | .025 |
| Asian | 1.105 | .704 | .727 | .239 |
| Multiple/Other | 1.493 | .052 | 1.455 | .086 |
| Hispanic | .730 | .091 | .923 | .675 |
| College Graduate (Bachelor’s+) | 1.161 | .182 | 1.136 | .288 |
| Republican | .512*** | .000 | .542*** | .000 |
| Public Insurance | .778* | .031 | .743* | .018 |
| High Health Literacy | 1.689*** | .000 | 1.908*** | .000 |
| Clinical work | .387** | .003 | .471* | .015 |
| Admin work | .393** | .004 | .244*** | .000 |
| Past-year hospital admission | .872 | .265 | .632*** | .000 |
| Poor Health | .980 | .938 | 1.097 | .736 |
| Mistreatment in Health care | 1.127 | .319 | 1.184 | .191 |
| Rural | 1.091 | .460 | 1.201 | .152 |
| Northeast | 1.000 |  | 1.000 |  |
| Midwest | .858 | .376 | .991 | .960 |
| South | .989 | .945 | 1.037 | .829 |
| West | 1.009 | .963 | 1.022 | .912 |
| Constant | .789 | .274 | 1.164 | .507 |
| ***p<.001; **p<.01; *p<.05  Reference groups are: Male gender, age 18–39 years, White race, non-Republican political affiliation, private insurance, low health literacy, no employment in health care, no past-year hospital admission, self-reported good health, non-rural residence, and Northeast region residence. | | | | |

*Figure 1 (Appendix) – Survey preamble framing conditions*

*
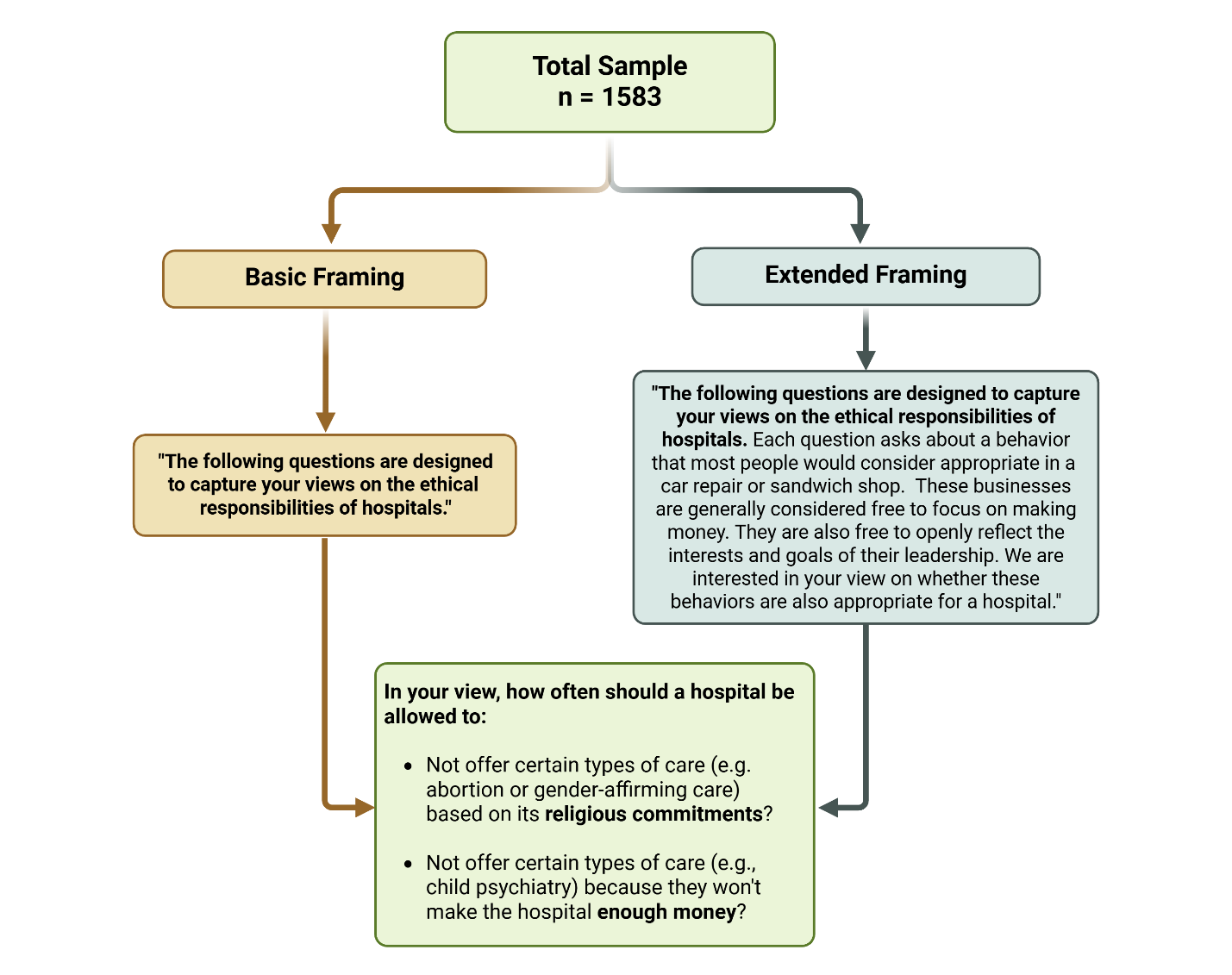
*
