# Supplementary figures and images for: Public views on religious and financial care restrictions in hospitals
Source: Health Aff Sch. 2026 Jan 25;4(2):qxag015. doi: 10.1093/haschl/qxag015 (PMC12869792; doi:10.1093/haschl/qxag015)

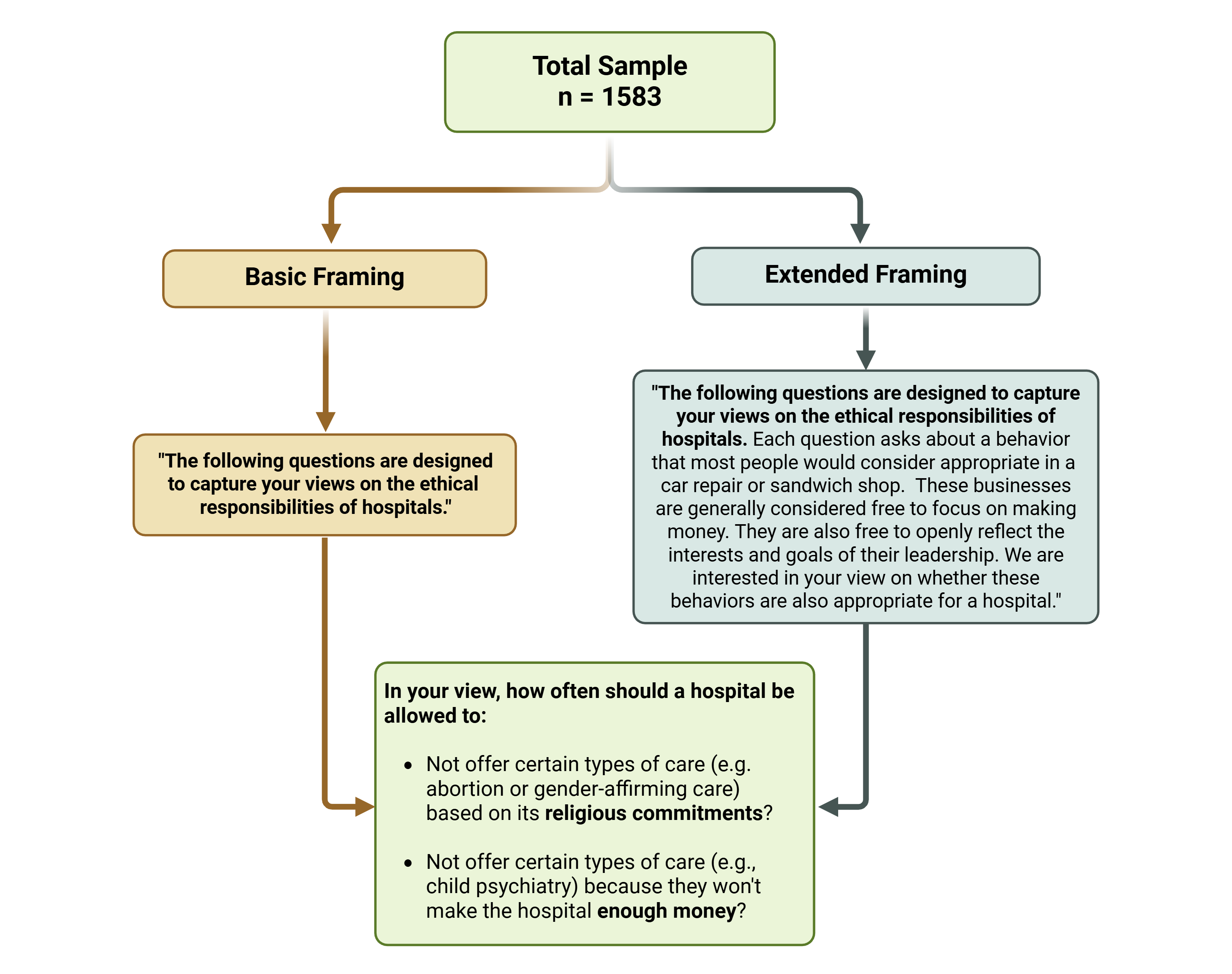

Supplement: qxag015_Supplementary_Data [file qxag015_supplementary_data.zip › Hospital Public Perceptions Figure 1.png]
